# Clinical trials in cancer

**DOI:** 10.1038/bjc.2011.137

**Published:** 2011-05-10

**Authors:** J A Green

**Affiliations:** 1 Senior Lecturer in Medical Oncology, Department of Molecular and Clinical Cancer Medicine, University of Liverpool, Liverpool, L69 3BX, UK

Clinical trials have evolved from early observational studies into accepted stages of development culminating in the phase III randomised clinical trial. The Cochrane collaboration has for many years sought to promote the quality of clinical trials, and developed methodology for meta-analysis, to which NICE has added health economic evaluation to make its cost effectiveness assessments, the final stage before implementation of research into clinical practice. It clearly makes sense for the trial population to reflect as far as possible the wider group in which the therapies will ultimately be used, and Stead *et al* (2011) in this issue of the journal report on 10 years experience of the National Cancer Research Network in promoting trials in the UK. However, for many rare tumours that now include subtypes of existing histopathological categories of cancer defined by molecular criteria, there are fresh challenges in evaluation of the available data, and it is noteworthy that the emphasis will change to improve support in those areas.

The stimulus for change in the UK was a government report in 1999 indicating poor cancer survival figures compared with Europe, and one of several initiatives was a comprehensive network of infrastructure support for clinical trials in cancer, starting in England in 2001 and later extending to the devolved nations (Scotland, Wales and Northern Ireland). Since 2001 many EU countries have established national organisations to promote clinical research, but none to date on the scale of the UK initiative in providing core infrastructural support for trials. The National Cancer Research Institute was created as a consortium of government and charitable funders in the UK, which was linked to funding of tumour group-specific committees. Representation was based on trial recruitment as well as geographical spread and led to the creation of a national portfolio of phase II and III trials.

The traditional model was that industry directly sponsored and ran registration studies, and provided academic grants to enable co-operative groups to implement a broader range of supporting studies across a range of tumour types. In Europe academic trial funding includes coordinated agencies such as the FP7 programme, as well as individual governments and charities providing grant support for peer reviewed studies, and co-operative groups which evolved around individual tumour-related organisations, in some cases growing to become global in scope. The EORTC went a stage further by creating additional generic groups, including some which were at the forefront of translational research such as receptors, pharmacokinetics and molecular mechanisms. Most groups operated on the basis of financial reimbursement per enrolled patient, and the UK model subsequently evolved to a hybrid system for more complex trials.

## Has this investment been successful?

The answer is a definite yes in some areas, not all of which could be foreseen at the outset, although in others the jury is still out. There have been clear overall achievements, and the numbers speak for themselves with the target of doubling the recruitment to clinical trials to 7.5% of incident cancer cases met in record time across the country, and 50% of NHS Trusts achieving a 10% target. A greater number of patients were recruited than through NCI-sponsored studies in 2007. Comparisons with the EORTC are less valid as there are a number of other European organisations carrying out phase II–III trials. There was also a reduction in the number of trials failing to meet their target. Perhaps the greatest achievement, however, was to stimulate a clinical research ethos across all NHS Trusts in the United Kingdom. Subsequent developments included the incorporation of research as an integral part of the work of all NHS hospitals supported by contract-based funding.

The process was not without its growing pains. The introduction of Research and Development units in NHS Trusts may have created ownership and a business model for research, at the expense of some uniformity and cooperation between individual organisations, whose varying interpretation of the regulations led to bureaucratic delays, a process which became a greater problem after the implementation of the EU clinical trials directive in 2001. Demand for research nurse time frequently grew to outstrip availability, particularly for follow-up, and was perhaps a necessary price of success. A little noticed addition to the programme in the later years was the incorporation of industry-sponsored trials, which sowed the seeds for later private public partnerships. The plethora of targets, compounds and advances in trial methodology in turn created an environment conducive to such interaction.

However, the answer to the main question whether overall cancer survival was improved as a result of the initiative is not clear. Proving cause and effect is always a challenge for broad health care interventions, and there are many facets to comparison of population-based survival assessments from registration criteria and completeness of data to confounding medical conditions and social deprivation factors. Even for individual cancers with a clear aetiological factor such as lung and cervical cancers, or those which are exquisitely sensitive to treatment such as testicular cancer, it takes many years for universal acceptance that any survival gain is clearly related to the intervention. The Department of Health update on the Cancer Reform Strategy published earlier this year makes no reference to promoting clinical trial activity, and concentrates on improving earlier diagnosis and uniform treatment access across the country, without challenging the historical basis of NHS funding.

## So what of the future?

Tissue sample collection is becoming part of the majority of clinical trials, which increasingly involve biomarker evaluation as a secondary or exploratory endpoint. Almost universal in phase I and II studies, it is now clear that molecular profiling in some form will be integral to many phase III studies as well. The cost of next generation sequencing technologies, widely expected to replace many current biomarkers, is falling at a rate, which will bring it into the range of a PET scan in the foreseeable future. Almost half of the NHS cancer networks are associated with Experimental Cancer Medical Medical Centres (ECMCs), which concentrate on phase I and II studies, and together with the Cancer Research Centres bring together expertise from industry and the Universities.

The improving outcomes strategy also highlights important programmes such as the Stratified Medicines Innovation Platform (https://ktn.innovateuk.org/web/ stratified-medicines-innovation-platform), which will provide crucial pilot data on gene sequencing for six selected tumours. Combined with a funding structure for quality-controlled molecular diagnostic testing, this would allow the generation of pre-stratified cohorts of patients for clinical trials. Upscaling that initiative to a national programme to cover a broader range of cancers and centres would be a logistical as well as a financial challenge. However, the achievements of the NCRN initiative in building cooperation between the government, national charities and the private sector provide a model from which to build the vision of personalised cancer medicine (Gonzalez-Angelo *et al*, 2010).

The Academy of Medical Sciences report on the EU directive may lead to more efficient trial approval across the country, with the creation of the Health Technology Authority (Rawlins, 2011). Web-based pathology review, and standardisation or centralisation of molecular analyses will minimise the variation of both inclusion and assessment criteria, and facilitate international collaboration. The involvement of industry is important, and may be fostered by expansion of joint training and exchange of staff. A further mark of success is that the comprehensive research networks have extended the model of national clinical research coordination to areas outside cancer, including diabetes, stroke and mental health.

One gap in the strategy that has received insufficient attention is communication with patients and their families. Molecular profiling may lack the instant appeal of a scan, but will characterise cancers with a much greater sensitivity and increasing specificity. An informed public will be more likely to participate in research than a suspicious one, and there is perhaps no better time to spend a modest proportion of the investment on education of an increasingly multicultural society on new developments in biomedical technology.
